# Characterization of 3D Printed Metal-PLA Composite Scaffolds for Biomedical Applications

**DOI:** 10.3390/polym14132754

**Published:** 2022-07-05

**Authors:** Irene Buj-Corral, Héctor Sanz-Fraile, Anna Ulldemolins, Aitor Tejo-Otero, Alejandro Domínguez-Fernández, Isaac Almendros, Jorge Otero

**Affiliations:** 1 Department of Mechanical Engineering, School of Engineering of Barcelona (ETSEIB), Universitat Politècnica de Catalunya, Av. Diagonal 647, 08028 Barcelona, Spain; aitor.tejo@upc.edu (A.T.-O.); alejandro.dominguez-fernandez@upc.edu (A.D.-F.); 2 Unitat de Biofísica i Bioenginyeria, Facultat de Medicina i Ciències de la Salut, Universitat de Barcelona, 08036 Barcelona, Spain; hector.sanz.fraile@hotmail.com (H.S.-F.); anna.ulldemolins@ub.edu (A.U.); isaac.almendros@ub.edu (I.A.); 3 CIBER de Enfermedades Respiratorias, 28029 Madrid, Spain

**Keywords:** steel-filled PLA, FFF, scaffold, grid structure, cell culture

## Abstract

Three-dimensional printing is revolutionizing the development of scaffolds due to their rapid-prototyping characteristics. One of the most used techniques is fused filament fabrication (FFF), which is fast and compatible with a wide range of polymers, such as PolyLactic Acid (PLA). Mechanical properties of the 3D printed polymeric scaffolds are often weak for certain applications. A potential solution is the development of composite materials. In the present work, metal-PLA composites have been tested as a material for 3D printing scaffolds. Three different materials were tested: copper-filled PLA, bronze-filled PLA, and steel-filled PLA. Disk-shaped samples were printed with linear infill patterns and line spacing of 0.6, 0.7, and 0.8 mm, respectively. The porosity of the samples was measured from cross-sectional images. Biocompatibility was assessed by culturing Human Bone Marrow-Derived Mesenchymal Stromal on the surface of the printed scaffolds. The results showed that, for identical line spacing value, the highest porosity corresponded to bronze-filled material and the lowest one to steel-filled material. Steel-filled PLA polymers showed good cytocompatibility without the need to coat the material with biomolecules. Moreover, human bone marrow-derived mesenchymal stromal cells differentiated towards osteoblasts when cultured on top of the developed scaffolds. Therefore, it can be concluded that steel-filled PLA bioprinted parts are valid scaffolds for bone tissue engineering.

## 1. Introduction

Additive manufacturing (AM) is a group of techniques in which three-dimensional structures are manufactured layer-by-layer in an automated way. It offers several advantages over the traditional subtractive or forming techniques: (1) it allows manufacturing complex shapes and even porous structures, (2) cheaper parts are produced if low-cost machines are employed; (3) it implies material, waste, and energy savings. Within the AM field, there are seven different categories [1]: binder jetting (BJ) [2], directed energy deposition (DED) [3], material extrusion (includes FFF—Fused Filament Fabrication and DIW—Direct Ink Writing) [4,5], material jetting (MJ) [6], powder bed fusion (PBF) (includes SLM—Selective Laser Melting and SLS—Selective Laser Sintering) [7], sheet lamination [8], and vat photopolymerization (includes SLA—stereolithography and Digital Light Processing (DLP) printing, as well as volumetric 3D printing) [9,10].

Within the different AM techniques, FFF is one of the most widely used technologies for rapid prototyping within the biomedical field, as it presents several advantages in terms of costs and the range of materials that can be used [11]. Also known as FDM (Fused Deposition Modelling), FFF uses a continuous filament of a thermoplastic material such as PolyLactic Acid (PLA) to build complex 3D structures in an automated way. One of the main disadvantages of FFF technology is the difficulty to ensure the correct bonding between layers [12]. The FFF 3D printing technique was patented in 1989 [13] and it bloomed up after its patent expired in 2009. After the technique became available to the general public, it has been used in different fields: automation, aeronautics, medicine, etc. Regarding the biomedical area, different applications could be highlighted, such as implants [14,15], 3D surgical planning prototypes [16,17], scaffolding [18,19], and regeneration of tissues [20].

There are numerous materials in the market for FFF 3D printing, for example acrylonitrile butadiene styrene (ABS) or Nylon, being PLA one of the most widely used, both alone and in combination with other materials such as wood, metals, or ceramics. Very little data have so far been published on systematic studies regarding the use of metal-filled filaments, since selecting compatible filler materials for the sake of improving the performance of polymeric composite materials is a difficult task [21]. In the present study, copper-, bronze-, and steel-filled PLA filaments are studied. Copper has an excellent heat and electric conductivity, it is easy to machine, bio-fouling resistant, and corrosion resistant [22]. Bronze alloy consists primarily of Cu, commonly with between 12 and 12.5% of Sn. It is a ductile alloy. Stainless steel is made of iron with typically a few tenths of carbon percentage, and with anti-corrosion elements such as Ni or Cr. It has high tensile strength, high corrosion resistance, and high biocompatibility. Therefore, it is used in a wide range of biomedical applications such as prostheses.

Regarding the mechanical properties of the metal-filled filaments, in some cases, increasing the metal content reduces the tensile strength and increases the thermal conductivity of the composite material studied. For example, Mohammadizadeh et al. [23] manufactured PLA filaments that contained copper, bronze, stainless steel, high carbon iron, and aluminum powders. They stated that the mechanical proper4ies of copper-filled-PLA were worse than those of PLA 3D printed parts. Additionally, they showed that the larger the layer height was, the lower the tensile strength, elastic modulus, and yield stress were. On the contrary, in different works, the mechanical properties were observed to increase when adding metals to the base polymer. Liu et al. [24] found that ceramic, copper, and aluminum-based PLA composite parts had similar or even superior mechanical properties when compared to bare PLA-made parts. Fafenrot et al. [25] developed polymer-metal materials 3D printed by FFF and concluded that the mechanical properties were similar to those of the PLA parts. On the other hand, there are other available options for the polymer matrix such as the use of ceramics. For instance, glass fiber-reinforced PLA can be employed in a wide range of applications, particularly in the biomedical, energy, and electronics industry [26]. In another example, Mahmoud et al. [27] studied the incorporation of two carbon fillers into the polypropylene: carbon nanotubes and synthetic graphite. The results showed that graphite-filled composites are more conductive than carbon nanotubes-filled composites. The flexural and tensile strength for both composites increased with the increase in the filler materials weight percentage. Later, the same authors [28] showed that flame-retardant MPP (melamine polyphosphate) had remarkable effects on the mechanical properties of the LLDPE (low-density polyethylene) composites. Five weight percentages of MPP were embedded into LLDPE, ranging from 5 to 30 wt%. It was concluded that the Young’s modulus increased, and the tensile break strength and the tensile yield strength increased monotonically with the increase in MPP content.

The addition of metal components to the polymers used in FFF printing opens a new world in different fields such as bioengineering, but more knowledge needs to be obtained on the optimization of the production of these biomaterials for the fabrication of novel scaffolds. Although there are some studies about cell growth on 3D printed ceramic zirconia toughened alumina (ZTA) scaffolds [29], few and non-concluding studies have been done with metal-filled polymeric materials. Moreover, previous studies have not focused on the biological response of cells in metal-PLA 3D printed parts, as cells were cultured on metal-based scaffolds, such as titanium 3D printed bases [30]. On the other hand, several studies have evaluated the cytocompatibility of 3D printed iron-based scaffolds for bone regeneration [11,31,32] so we hypothesize that composite polymers incorporating metals will be appropriate for cell growth, since metals and alloys have been used extensively as bone substitutes [33,34]. These iron-based alloys have better mechanical properties than those based on lighter materials, such as magnesium.

Porosity is another key parameter that must be taken into consideration during the design and synthesis of a biomaterial [35]. The 3D printed porous materials should ideally fulfil conditions such as biocompatibility, noninflammatory response, tunable biodegradability, appropriate mechanic properties, defined pore structure, and, above all, promote a health improvement [36].

This work presents the characterization of three metal-reinforced PLA biomaterials for 3D printing biomedical scaffolds regarding porosity, surface roughness and cell culture. For that purpose, first the surface of the parts was analyzed, and the line spacing was measured. Surface roughness was then measured on the upper surface of the specimens. Regarding biological characterization, human-derived bone marrow mesenchymal stromal cells (hBM-MSC) were cultured on the composite scaffolds to assess their biocompatibility and the effect of the 3D printing scaffolds on determining cell fate, specifically in osteogenic differentiation.

## 2. Materials and Methods

### 2.1. Materials

The materials used in the present study were metal-filled PLA filaments of 2.85 mm diameter manufactured by ColorFabb (Belfeld, Netherlands). The specific materials used were: (1) steel-filled PLA, (2) bronze-filled PLA, and (3) copper-filled PLA. As stated by the manufacturer, these materials were developed for aesthetic purposes and need a polishing treatment after being printed if a brilliant appearance is to be required. All reagents were purchased from Sigma-Aldrich (Saint Louis, MO, USA), unless specified otherwise.

### 2.2. 3D Printing Process

**Figure 1:**
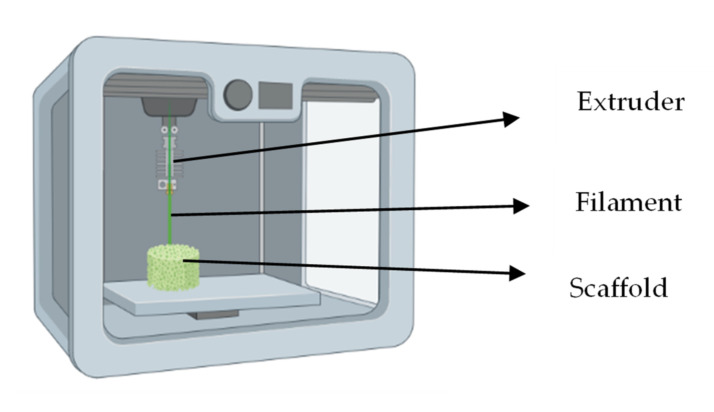
FFF 3D printer scheme.

Parts were additively manufactured by using a Sigma R19 3D printer (BCN3D Technologies, Gavà, Spain). The 3D printing parameters are presented in Table 1. Figure 1 shows a scheme of an FFF 3D printer. Cura BCN3D software was used to generate the G-code that is required to print the parts.

**Figure 2:**
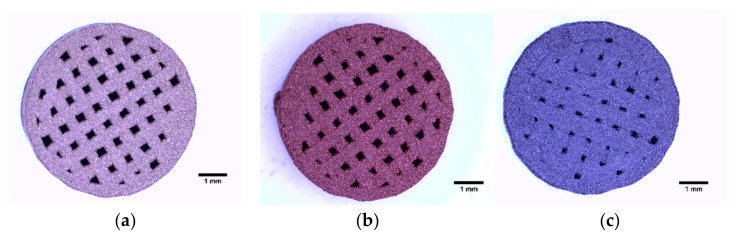
Disk-shaped 3D printed samples with line spacing of 0.7 mm of (**a**) bronze-filled PLA, (**b**) copper-filled PLA, (**c**) steel-filled PLA. Scale bars correspond to 1 mm.

Disk-shaped samples of 6 mm in diameter and 2 mm in height were manufactured. The linear infill pattern was selected, using three different line spacing values: 0.6 mm, 0.7 mm, and 0.8 mm (Figure 2). The shell width was set to 0.4 mm and the bottom width was set to 1.2 mm. No top layer was used.

### 2.3. Pictures

**Figure 3:**
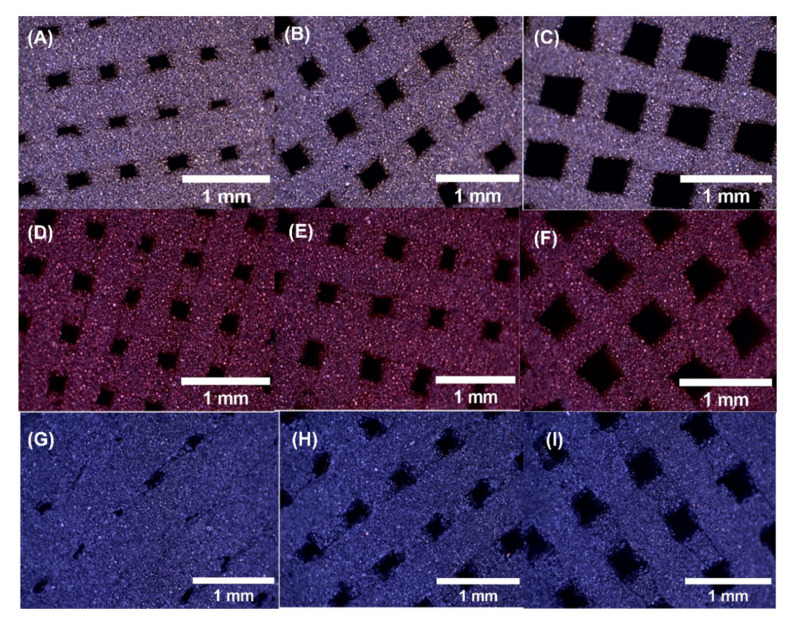
Surface of the samples with 16× magnification: (**A**) bronze-filled 0.6 mm, (**B**) bronze-filled 0.7 mm, (**C**) bronze-filled 0.8 mm, (**D**) copper-filled 0.6 mm. (**E**) copper-filled 0.7 mm, (**F**) copper-filled 0.7 mm, (**G**) steel-filled 0.6 mm, (**H**) steel-filled 0.7 mm, (**I**) steel-filled 0.8 mm. The scale bars correspond to 1 mm.

Pictures of the 3D printed scaffolds were obtained by using a Leica S8AP0 binocular magnifier (Leica Camera AG, Wetzlar, Germany) with 8× (Figure 2) and 16× (Figure 3) magnification, respectively.

### 2.4. Porosity

The porosity of the disk samples was quantified from the images of the cross section of the scaffolds, assuming that the length of the pores corresponds to the length of the sample and using Equation (1):

(1) $$Pt=\frac{Vp}{Vt}$$

where *Pt* is the porosity, *Vp* the pore volume, and *Vt* the total volume of the scaffold. The software used for cross-sectional images quantification was ImageJ.

### 2.5. Roughness

Roughness was measured with a Talysurf 2 contact roughness meter from Taylor Hobson Ltd., Leicester, UK. A diamond tip was used with tip angle of 90° and tip radius of 2 µm. The measuring force was 0.8 mN and speed was 0.5 mm/s. A Gaussian filter was employed. A cut-off value of 0.8 mm was used according to ISO 4288 [37]. Total sampling length was 4.8 mm (6 × 0.8 mm). Roughness was measured on the upper surface of the disks, along the generatrices of the filaments in the two perpendicular directions, in order to assess if there were differences regarding their surface finish. As an example, the steel-filled samples were measured with line spacing 0.6 and 0.7 mm. Two different samples were measured for each line spacing value.

The roughness parameters that were analyzed in this present study are:

− Arithmetical mean roughness value or arithmetical mean of the absolute values of the profile deviations from the mean line of the roughness profile (Ra) (Equation (2)), which is one of the most commonly employed parameters in industry;

(2) $$\mathrm{Ra}=\frac{1}{L}\int_{0}^{L}\left|Z\left(x\right)\right|dx$$

− Mean roughness depth or average maximum peak to valley of five consecutive sampling lengths of the profile within a sampling length (Rz);
− Kurtosis (Rku), which is a measure of the sharpness of the profile (Equation (3)); and:

(3) $$\mathrm{Rku}=\frac{1}{R_{q}^{4}}\left[\frac{1}{L}\int_{0}^{L}Z^{4}\left(x\right)\;dx\right]$$

− Skewness (Rsk), which measures the symmetry of the profile (Equation (4)).

(4) $$\mathrm{Rsk}=\frac{1}{R_{q}^{3}}\left[\frac{1}{L}\int_{0}^{L}Z^{3}\left(x\right)\;dx\right]$$

These parameters are defined in the UNE-EN-ISO 4287:1999 standard [38].

### 2.6. Human Bone Marrow-Derived Mesenchymal Stromal Cells Culture on the Developed Scaffolds

Primary human Bone Marrow-Derived Mesenchymal Stromal cells (hBM-MSCs, ATCC PCS-500-012, ATCC, Manassas, VA, USA) were expanded following the manufacturers’ instructions. Cells from passage 3–6 were used for all the experiments presented herein.

Printed parts were coated by incubating with rat tail-derived type I collagen at a concentration of 0.1 mg/mL for 30 min at 37 °C. hBM-MSCs were cultured on the scaffolds at a seeding density of 4 × 10^4^ cells/cm^2^ for 24 and 72 h. Cells seeded on uncoated parts were cultured in parallel. At the defined time points, cells were fixed with paraformaldehyde (PFA) 4% for further immunohistochemical imaging.

In a subsequent set of experiments, hBM-MSCs were cultured with a seeding density of 9.4 × 10^4^ cells/cm^2^ for 1, 4, and 7 days, respectively, on the different collagen-coated/uncoated samples and then fixed with PFA 4%.

Finally, to evaluate the impact of the developed scaffolds on the cell fate, hBM-MSCs were cultured with αMEM (12509069, Gibco, Waltham, MA, USA) 10% FBS (Gibco) on the steel-filled PLA scaffolds with 0.6, 0.7, and 0.8 mm line spacing, respectively, with a seeding density of 1.3 × 10^4^ cells/cm^2^. Control cells were cultured in parallel on conventional culture plates. After 21 days, the cells were fixed with PFA 4% for further analysis.

### 2.7. Immunohistochemical Analysis

After PFA fixation, samples were permeabilized with Triton 0.1%, blocked with 10% FBS solution, and incubated overnight at 4 °C with primary antibodies (anti-hOsteocalcein 967801, R&D Systems, Minneapolis, MN, USA) and subsequently, incubated for 2 h at 37 °C with the secondary Alexa 488 anti-rabbit antibody for differentiation studies. For morphology analysis, nuclei were stained with NucBlue (Thermo Scientific, Waltham, MA, USA) and actin cytoskeleton with phalloidin (Thermo Scientific, Waltham, MA, USA). Images were acquired with a Nikon D-Eclipse Ci confocal microscope (Nikon, Tokyo, Japan) with 10× and 20× Plan Apo objectives (Nikon).

## 3. Results and Discussion

### 3.1. 3D Printed Samples

Figure 3 shows the pictures of bronze-filled, copper-filled, and steel-filled samples manufactured with 0.6, 0.7, and 0.8 mm line spacing, respectively. Achieving 0.6 mm line spacing was more difficult than 0.8 mm, because of smaller pores. Despite that, appropriate scaffolds were achieved for the three different materials.

Additionally, scaffolds for biomedical applications should have a porous architecture. This porosity provides the necessary environment for promoting cell migration, proliferation, etc. [39].

### 3.2. Porosity

**Figure 4:**
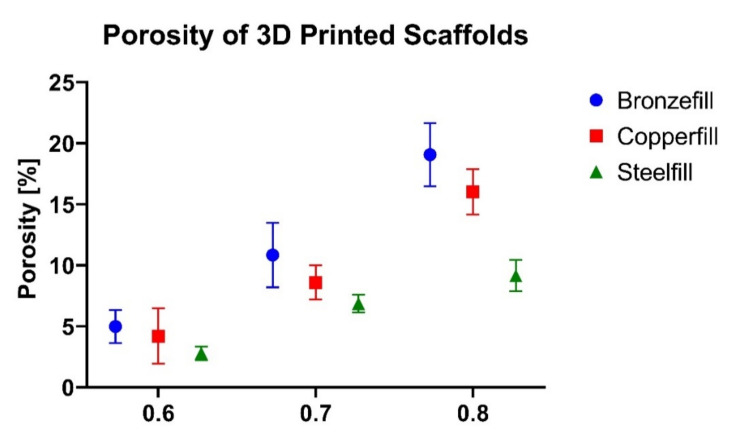
Porosity of the 3D printed scaffolds: bronze-filled, copper-filled and steel-filled. *N* = 3.

As shown in Figure 4, the higher the line spacing, the higher the porosity of the 3D printed scaffolds is. Among the different scaffolds, for a certain line spacing value, most porous scaffolds are the bronze-filled ones, followed by the copper-filled ones, although they were manufactured with the same 3D printing conditions:

### 3.3. Roughness

Table 2 presents the roughness results for both the internal and the external surfaces of the 3D printed samples. One measurement was performed on each surface.

**Figure 5:**
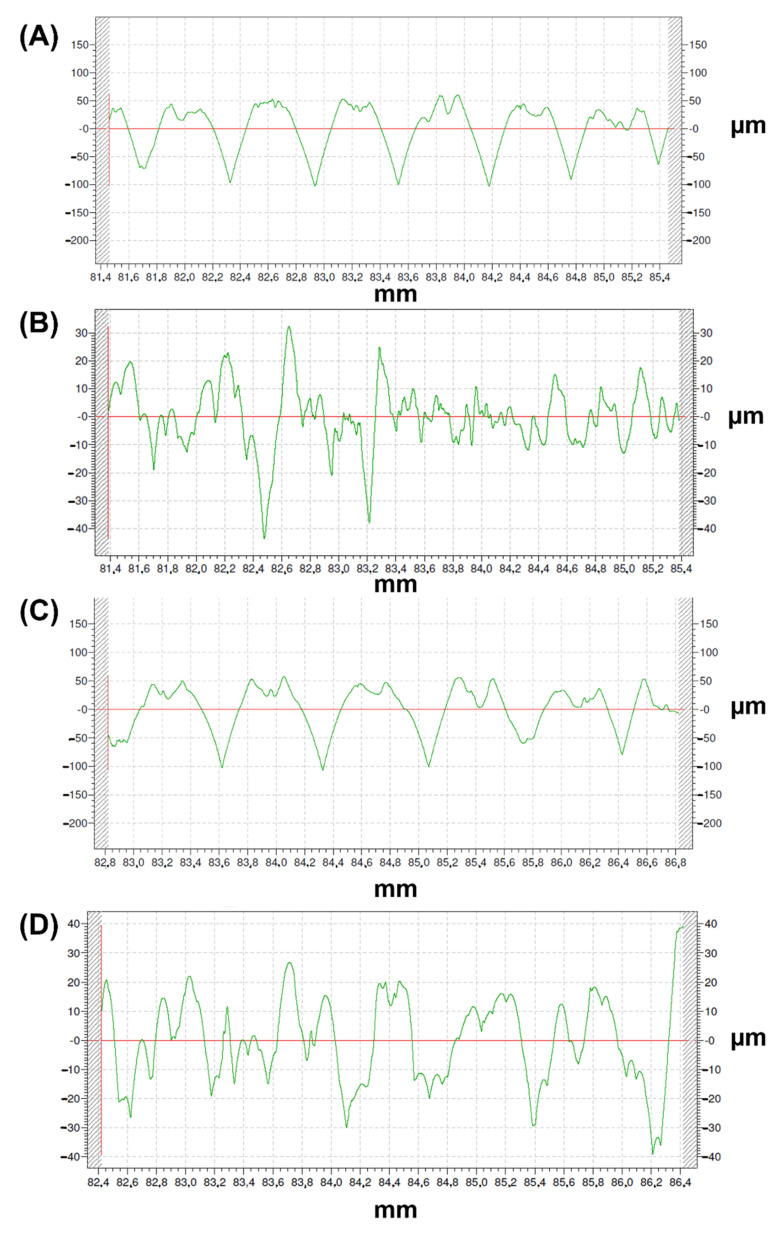
Roughness profiles of the steel-filled 3D printed samples. (**A**) 0.6 mm external roughness (first layer). (**B**) 0.6 mm internal roughness (second layer). (**C**) 0.7 mm external roughness (first layer). (**D**) 0.7 mm internal roughness (second layer).

Figure 5 depicts the roughness profiles of samples with a line spacing of 0.6 and 0.7 mm, on the external (first) and internal (second) layers, respectively, starting from the top of the part. Higher Ra values were obtained on the external (first) layer (Table 2 and Figure 5a,c) than on the internal (second) layer (Table 2 and Figure 5b,d). On the external layer, slightly higher Ra values (up to 35.04 µm) were found for line spacing 0.7 mm than for line spacing 0.6 mm (up to 25.60 µm). The Rz parameter shows a similar trend than Ra.

Rku values around 3 were found in all cases, corresponding to a normal distribution of the roughness heights in each profile. On the external layer, slightly negative Rsk values were obtained, corresponding to longer valleys than crests. On the contrary, on the second layer Rsk values are close to 0, corresponding to symmetric profiles.

The external or first layers show more regular roughness profiles (Figure 5a,c) than the internal or second layers (Figure 5b,d).

### 3.4. Human Bone Marrow-Derived Mesenchymal Stromal Cells Cultured on the Developed Scaffolds

**Figure 6:**
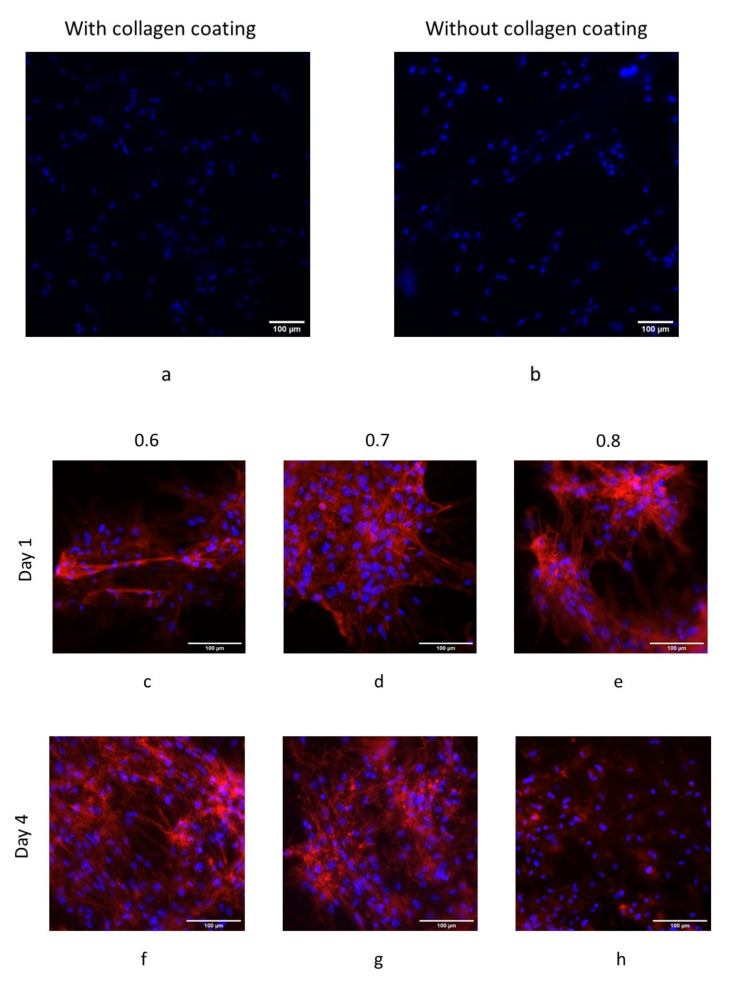

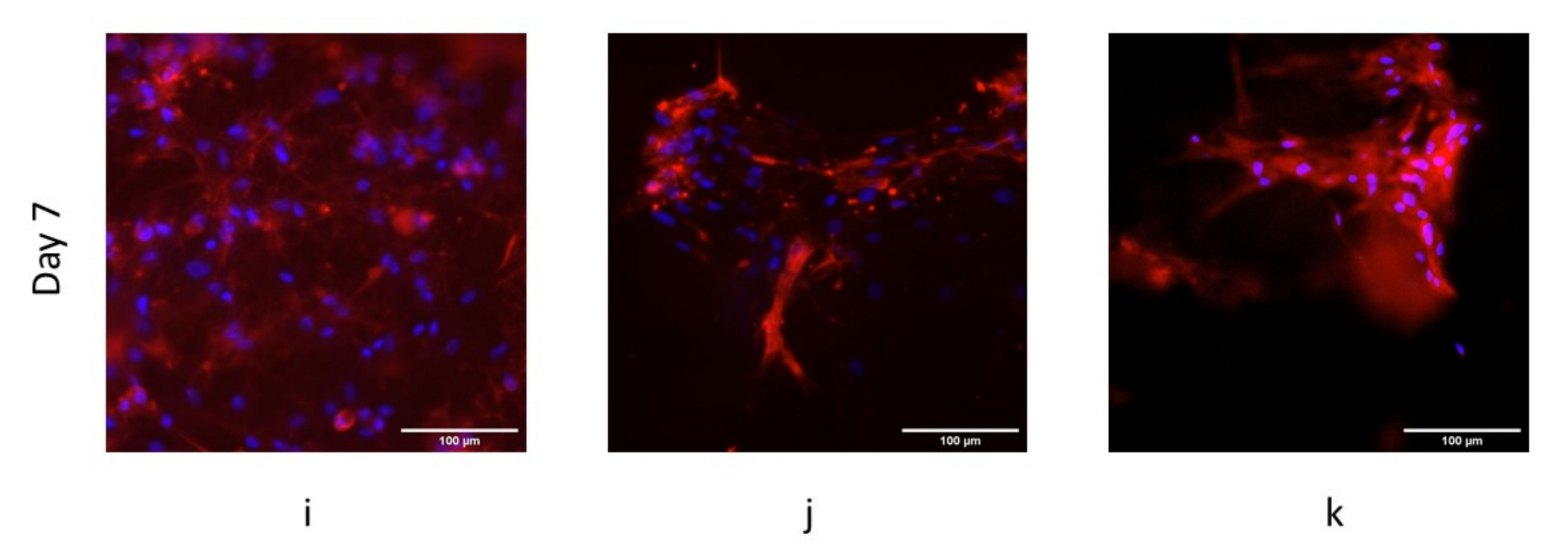
(**a**,**b**) Adhesion test to PLA with and without collagen coating. (**c**–**e**) Viability test at day 1 of cell culture. (**f**–**h**) Viability test at day 4 of cell culture. (**i**–**k**) Viability test at day 7 of cell culture.

hBM-MSCs showed good adhesion to both collagen-coated and untreated 3D printed steel-filled PLA samples. Cells cultured for both 24 and 72 h were well-adhered to the 3D printed PLA composites (Figure 6a,b), with no observed differences between both conditions.

Steel-filled PLA samples showed a very good cytocompatibility, especially on 0.6 and 0.7 line spacing (Figure 6c,d,f,g,i,j). On the contrary, copper-filled and bronze-filled PLA presented higher cytotoxicity since there were no cells adhered to the scaffolds after 24 h of culture (data not shown).

Steel-filled scaffolds showed high biocompatibility, unlike copper-filled and bronze-filled materials. This is in concordance with Kuroda et al. [39]. Additionally, the best biological behavior was found with the lowest porosity achieved (0.6 mm line spacing). This is in accordance with data presented by Chen et al. [40], who concluded that samples with 30% porosity exhibit the best biocompatibility, which were the lowest porosity scaffolds of their research.

The different scaffolds manufactured by means of FFF showed to have different behavior. As mentioned, bronze-filled as well as copper-filled scaffolds presented high cytotoxicity since there were no cells adhered to the scaffolds after 24 h of culture.

It is interesting to highlight that there were no differences observed when a specific protein coating was used in the parts. Cells form specific adhesions to the collagen protein while they are expected to form unspecific adhesions to uncoated materials. From the experiments presented herein, it can be concluded that steel-filled PLA promotes the formation of unspecific adhesion in MSCs, while this is not happening with copper- or bronze-filled polymers.

### 3.5. hBM-MSCs Differentiated towards Osteoblasts When Cultured on the Developed Scaffolds

**Figure 7:**
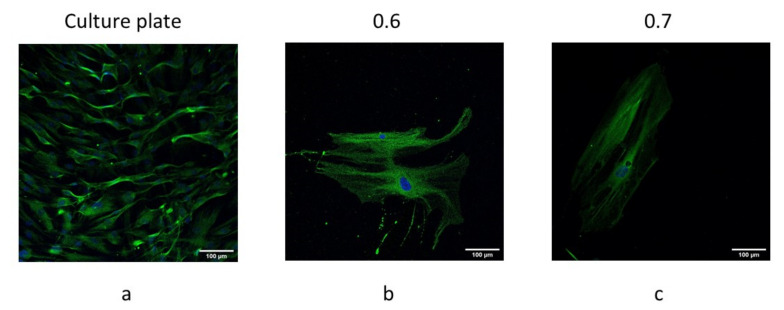
Osteocalcin detection by immunofluorescence in hBM-MSCs (at day 21) cultured on (**a**) conventional culture conditions, steel-filled PLA scaffolds of (**b**) 0.6 mm and (**c**) 0.7 mm line spacing. Osteocalcin (green) and nucleus (blue). Scale bars correspond to 100 µm.

hBM-MSCs cultured on the steel-filled 3D printed scaffolds (0.6 and 0.7 mm line spacing) without osteogenic supplements showed the presence of osteocalcin after 21 days of culture (Figure 6). Moreover, cells exhibited a broad spreading area compared with those cultured under conventional culture conditions (Figure 7).

The images show the classical spindle-like shape of MSCs, so it seems that the steel-filled 3D printed structures are a suitable scaffold for cell culturing. The enlarged phenotype is similar to differentiated osteoblasts when cultured on rigid substrates [41,42,43].

## 4. Conclusions

In the present work, results are presented for the cell growth of stem cells on metal-filled PLA composites that were printed with a grid structure by means of the FFF technique. Three different composites were tested: bronze, copper, and stainless steel, respectively. The main conclusions are as follows:

− Given a certain line spacing, higher porosity was observed for the copper-filled scaffolds than for the bronze-filled scaffolds and the steel-filled scaffolds, although they were 3D printed with similar printing conditions;
− Steel-filled composite showed important cell growth, both with and without protein coating, so it is promoting the formation of unspecific adhesions in MSCs;
− Neither bronze-filled nor copper-filled composites favored cell growth, so they cannot be considered to be biocompatible;
− When considering steel-filled composite, line spacing of 0.6 and 0.7 mm provided the best results, while line spacing of 0.8 mm is not recommended.

In future work, the effect of the use of other infill patterns on both cell growth and the mechanical strength of the structures will be addressed.

## Figures and Tables

**Table 1:** 3D printing parameters.

Variable	Value
Infill pattern	Linear
Layer height (mm)	0.15
Nozzle diameter (mm)	0.4
Print speed (mm/s)	7
Extrusion multiplier (%)	100
Temperature (°C)	190

**Table 2:** Roughness on the external and internal layer of 3D printed steel-filled samples.

Line Spacing	Sample	External Roughness				Internal Roughness			
		Ra (µm)	Rz (µm)	Rku	Rsk	Ra (µm)	Rz (µm)	Rku	Rsk
0.6 mm	1	25.36	128.90	3.12	−1.00	7.99	38.67	2.91	0.24
	2	25.60	129.11	3.14	−1.03	7.97	37.16	3.29	0.44
0.7 mm	1	32.38	142.63	2.54	−0.73	12.13	52.91	2.60	−0.09
	2	35.04	158.71	2.73	−0.80	15.68	78.31	2.89	0.04

## Data Availability

Data supporting the findings of this study are available from the corresponding authors upon reasonable request.
